# PypKa server: online **p*K*_a_** predictions and biomolecular structure preparation with precomputed data from PDB and AlphaFold DB

**DOI:** 10.1093/nar/gkae255

**Published:** 2024-04-15

**Authors:** Pedro B P S Reis, Djork-Arné Clevert, Miguel Machuqueiro

**Affiliations:** BioISI – Instituto de Biossistemas e Ciências Integrativas, Faculdade de Ciências, Universidade de Lisboa, 1749-016 Lisboa, Portugal; Machine Learning Research, Bayer AG, Müllerstraße 178, 13353 Berlin, Germany; Machine Learning Research, Bayer AG, Müllerstraße 178, 13353 Berlin, Germany; Machine Learning Research, Pfizer, Berlin, Germany; BioISI – Instituto de Biossistemas e Ciências Integrativas, Faculdade de Ciências, Universidade de Lisboa, 1749-016 Lisboa, Portugal

## Abstract

When preparing biomolecular structures for molecular dynamics simulations, p*K*_a_ calculations are required to provide at least a representative protonation state at a given pH value. Neglecting this step and adopting the reference protonation states of the amino acid residues in water, often leads to wrong electrostatics and nonphysical simulations. Fortunately, several methods have been developed to prepare structures considering the protonation preference of residues in their specific environments (p*K*_a_ values), and some are even available for online usage. In this work, we present the PypKa server, which allows users to run physics-based, as well as ML-accelerated methods suitable for larger systems, to obtain p*K*_a_ values, isoelectric points, titration curves, and structures with representative pH-dependent protonation states compatible with commonly used force fields (AMBER, CHARMM, GROMOS). The user may upload a custom structure or submit an identifier code from PBD or UniProtKB. The results for over 200k structures taken from the Protein Data Bank and the AlphaFold DB have been precomputed, and their data can be retrieved without extra calculations. All this information can also be obtained from an application programming interface (API) facilitating its usage and integration into existing pipelines as well as other web services. The web server is available at pypka.org.

## Graphical Abstract

**Graphical Abstract:**
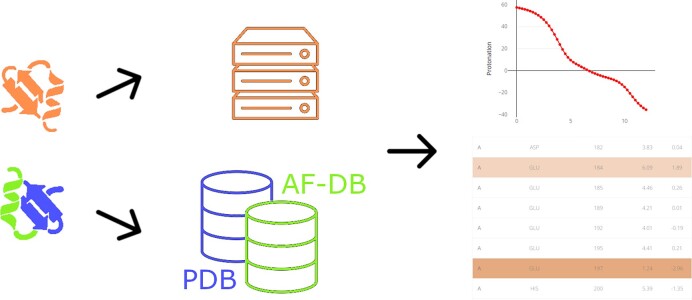

## Introduction

Before running popular molecular modelling methods like molecular dynamics (MD; e.g. GROMACS (1), NAMD (2), etc.), molecular docking (e.g. HADDOCK (3), AutoDock (4), etc.) or continuum electrostatics (e.g. APBS (5), DelPhi (6), etc.) it is necessary to prepare protein starting structures (experimental or models). It is often necessary to rebuild partially resolved side-chain atoms in protein X-ray structures and add missing hydrogens, which are generally absent. Furthermore, the conditions (like pH and ionic strength) at which the experimental structure was determined rarely coincide with the desired ones, which can affect significantly the ionization state of a protein (7,8). Thus, p*K*_a_ calculations are required to correctly place polar hydrogen atoms and to attain a representative protonation state at the user-defined conditions. p*K*_a_ estimations are also valuable for factoring in the electrostatic environment, thereby offering molecular insights through the final values.

There are already several tools available that allow users to prepare their structures and/or obtain p*K*_a_ estimations. In the PDB2PQR web server (9) it is possible to process pdb files into clean pqr files with direct integration with APBS to easily run electrostatics calculations. To determine the most abundant protonation states of titratable sites, an empirical p*K*_a_ predictor (PROPKA (10)) is used, and in this server, it is possible to inspect the log files produced to find the p*K*_a_ predictions. Other p*K*_a_ calculator web servers, like H++ (11) and DelPhiPKa (12), rely on the use of continuum electrostatics instead of statistical methods. Compared to PROPKA, these Poisson–Boltzmann-based predictors afford a more detailed description of the inter-atomic interactions at the cost of higher computational time. In H++, the p*K*_a_ and isoelectric point information is complemented with the generation of AMBER-compatible structures and topologies (with both implicit and explicit solvent) and there is even the option to neutralize the system with counterions. Also noteworthy are the IPC 2.0 and Proteome-pI 2.0 releases. In these online servers, users can input protein sequences and get p*K*_a_ values and isolectric points based on several different models as well as query isolectric points of 20k proteomes (13,14).

The initial (and often rate-limiting) step to studying biological systems with atomistic detail is to acquire a molecular structure. A common source is the Protein Data Bank (PDB) which at the present contains almost 200k experimentally determined structures (15). However, many of these structures correspond to the same protein and there is no structure available for most biologically occurring proteins. In the case of the human proteome, the most represented in the PDB, just 35% of proteins have a PDB entry and many of these are only partially solved (16,17). Homology modelling is often employed when a specific protein structure is not available, but there is a good degree of sequence similarity with other known structures. In recent years, machine learning (ML) alternative models have been proposed to predict 3D structures from sequences of amino acids with remarkable accuracy (18,18,19). Most notably, AlphaFold was applied to several proteomes and the predicted structures have been made available (17,20). These structures, similar to experimental ones, lack hydrogen atoms and require preprocessing before they can be used in a molecular modelling pipeline.

It is clear that the same PDB structures have likely been repeatedly prepared countless times by different research groups. To save computational resources and streamline structure preparation from the PDB, we have calculated the p*K*_a_ values on these structures using our continuum electrostatics-based p*K*_a_ predictor (PypKa (21)). With these p*K*_a_ values collected in the FAIR compliant (22) pKPDB database (23), it became possible to efficiently retrieve structures with representative protonation states. Since its publication, the pKPDB database has been extended to more than 100k structures obtained from the AlphaFold DB selection of model organisms (*C. albicans*, Zebrafish, Fruit Fly, *E. coli*, Mouse, Human, Rat and Yeast) and global health proteomes (*S. pneumoniae*, *H. pylori*, *M. leprae* and *N. gonorrhoeae*). In future, we plan on continuously adding more proteomes to the over 200k structures and 20M p*K*_a_ values available currently.

In this work, we present the PypKa server, an online web service to calculate p*K*_a_ values of proteins and prepare biomolecular structures for usage in downstream applications. The user may select a structure by its PBD identification code or UniProtKB accession number. In the query, if a previous result of the selected structure is found in the pKPDB, no calculation is required/performed, and the cleaned pdb file can be instantly accessed. The user can also upload a custom pdb file which will trigger a new p*K*_a_ calculation. Very large structures (over 1500 residues), which would be too time and resource-consuming for PypKa, can now be easily handled by pKAI (24). In this server, it is also possible to obtain input structures compatible with the most popular MD force fields, like CHARMM, AMBER and GROMOS. Furthermore, the p*K*_a_ values, and the isoelectric points, can be obtained from a REST API, making it easy to incorporate into the user’s own pipelines and services.

## Materials and methods

### Implementation

#### Front end

The PypKa server is a progressive web app powered by the React-based GatsbyJS framework. The standard web technologies HTML5, CSS3 and JavaScript were used, and most modern web browsers are supported. The Protein Data Bank Web Services is used to fetch information about the user-selected accession codes. The AlphaFold Protein Structure Database developed by DeepMind and EMBL-EBI is accessed to download the AlphaFold generated structures by their UniProtKB identifier. The communication with the back end is done mainly via a REST API, except for the submission results retrieval, which is done via Server-Sent Events to facilitate the real-time data transfer from the server. The titration plots shown on the results page are rendered by the plotly.js graphing library.

#### Back end

The back end is hosted in a Ubuntu 22.04 instance and can be divided into several components: a REST API service, an HTTP server, resources and jobs manager, and a relational database. The REST API and Server-Sent Events run on Python3.8 on top of an Nginx v1.14.0 server that handles the requests. We use Flask v2.0.1 for the API implementation and Tornado v6.1 for the WebSocket. Slurm v20.02.7 is used to manage the resources and PypKa jobs allocation efficiently. PypKa v2.10.0 (21) is the p*K*_a_ predictor being used with default settings unless otherwise specified by the user. pKAI v.1.2.0 (24) is the ML model used to accelerate the p*K*_a_ calculations in large systems. The pdbmender library v0.4.1, which uses the PDB2PQR (9) to reconstruct missing atoms and deal with different naming nomenclatures, handles the input structures preprocessing and also creates the output structures with the correct protonation states, estimated by the p*K*_a_ predictors. The pKPDB (23) with precomputed p*K*_a_ values, isoelectric points and titration curves is implemented in PostgreSQL v12.9.

#### Workflow

**Figure 1.**
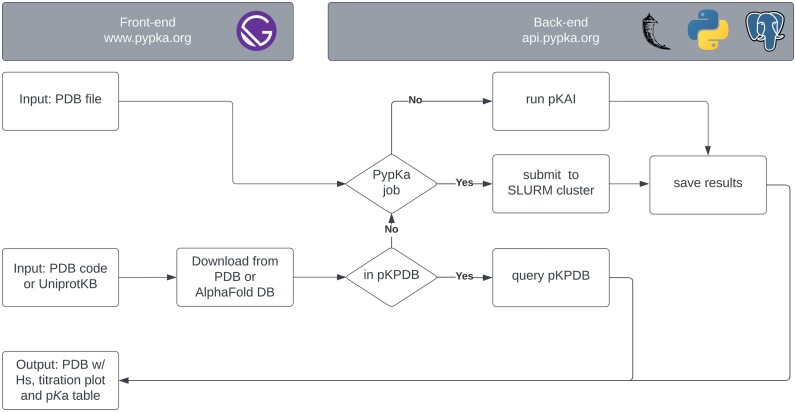
Overview of the PypKa web server workflow.

The PypKa server workflow is illustrated in Figure 1. At the front end, the user may submit a job using a pdb file, a PDB code, or a UniProtKB identifier. If a custom pdb file is uploaded, the user may select which p*K*_a_ predictor to use. By default, PypKa will be selected for structures with less than 1500 residues, and pKAI will be used for the remaining larger systems. While PypKa has shown to be highly scalable (21), it is still quite computationally expensive to run on these larger systems. Furthermore, a three-steps focusing procedure would need to be implemented to efficiently and accurately tackle such systems (25). The effectiveness of pKAI at replicating PypKa results and its remarkable efficiency (24) positions this method as an attractive alternative. If a PypKa prediction is selected, the job will be submitted to the Slurm cluster. After running the p*K*_a_ calculation, the results will be stored, returned to the front end, and presented to the user. If the user submits a job using a PDB or UniProtKB code, the pKPDB will be queried to check if it contains the corresponding results (true if default settings were used). If the query is successful, the results will be immediately returned to the user as there is no need to proceed with further calculations. In the opposite scenario, a new p*K*_a_ prediction will be performed using a structure downloaded from the PDB or AlphaFoldDB.

#### REST API

It is possible to use the PypKa server without interacting with the web UI. The REST API allows users and other services to easily integrate p*K*_a_ calculations into their existing pipelines. In the /pkas endpoint, users can retrieve p*K*_a_ values, the isoelectric point, and the titration curve of a protein. In some cases, the results may be obtained directly from the pKPDB (e.g. api.pypka.org/pkas/4LZT). In the event of a submission made with a protein code that has not been previously computed and stored in the pKPDB, a very fast pKAI calculation will take place and its results will be returned to the user request (e.g. api.pypka.org/pkas/P00698). In order to ensure fair usage we are enforcing a rate limit of 100 requests per hour. Users with applications that require higher usage are welcome to request an API key.

## Results

The PypKa server allows users to effortlessly run p*K*_a_ predictions and use them to prepare biomolecular structures for other molecular modelling methods. Although only proteins are titrated, nucleic acids, lipids, and ions can be included in the calculations and contribute as background charges. Three methods are offered to the user (PypKa, pKAI and pKAI+), each with its own set of pros and cons. PypKa is the only physics-based method and the only one that can include other molecules in the calculations. It is thus the most reliable but also the most time-consuming (see Table 1). As expected, the computational cost increases with the size of the protein and its number of titratable residues. pKAI is an ML model trained to reproduce PypKa predictions at a much lower computational cost. Therefore, it will be adopted when dealing with very large systems. pKAI+ is a variant of the pKAI that penalizes predictions with large p*K*_a_ shifts in an attempt to attenuate the effect of using a single (possibly unrepresentative) structure to describe the conformational ensemble of a protein. However, while pKAI+ yields a smaller error compared to pKAI or PypKa at predicting experimental p*K*_a_ values, the latter methods perform better at choosing the most representative protonation state at the physiological pH range (24). The input pdb file for the calculations may have been experimentally solved or obtained from computational methods, such as MD or homology modelling, and the nomenclature of several popular force fields (AMBER, CHARMM and GROMOS) is supported. The user may select a custom pdb file or an accession code from PDB or UniProtKB. In this case, and if the default parameters are used, a database with p*K*_a_ values and related quantities for 200k structures taken from the PDB and AlphaFold DB will be queried and immediately returned with no extra calculations performed.

**Table 1.** Typical user waiting times from job submission to final results

			Time to results (s)		
PDB Code	# Res.	# titr. Res.	PypKa	pKAI	pKPDB
1A1W	83	23	12 s	1.1 s	0.5 s
102L	163	39	18 s	1.2 s	0.5 s
16VP	311	65	43 s	1.3 s	0.5 s
15C8	430	101	1m07s	1.4 s	0.5 s
1A01	574	162	2m02s	1.6 s	0.5 s
1ACO	753	199	5m27s	1.9 s	0.5 s
1A6D	1005	280	10m44s	2.5 s	–
1A4Y	1166	308	16m08s	2.8 s	–
1CB5	1359	405	24m06s	3.6 s	–

The reported times assume there is no latency in the communication with the server and thus do not consider the user’s internet connection speed. PypKa runs are performed in parallel on 16 cores (Intel Xeon Silver 4214R, 2.40 GHz).

**Figure 2.**
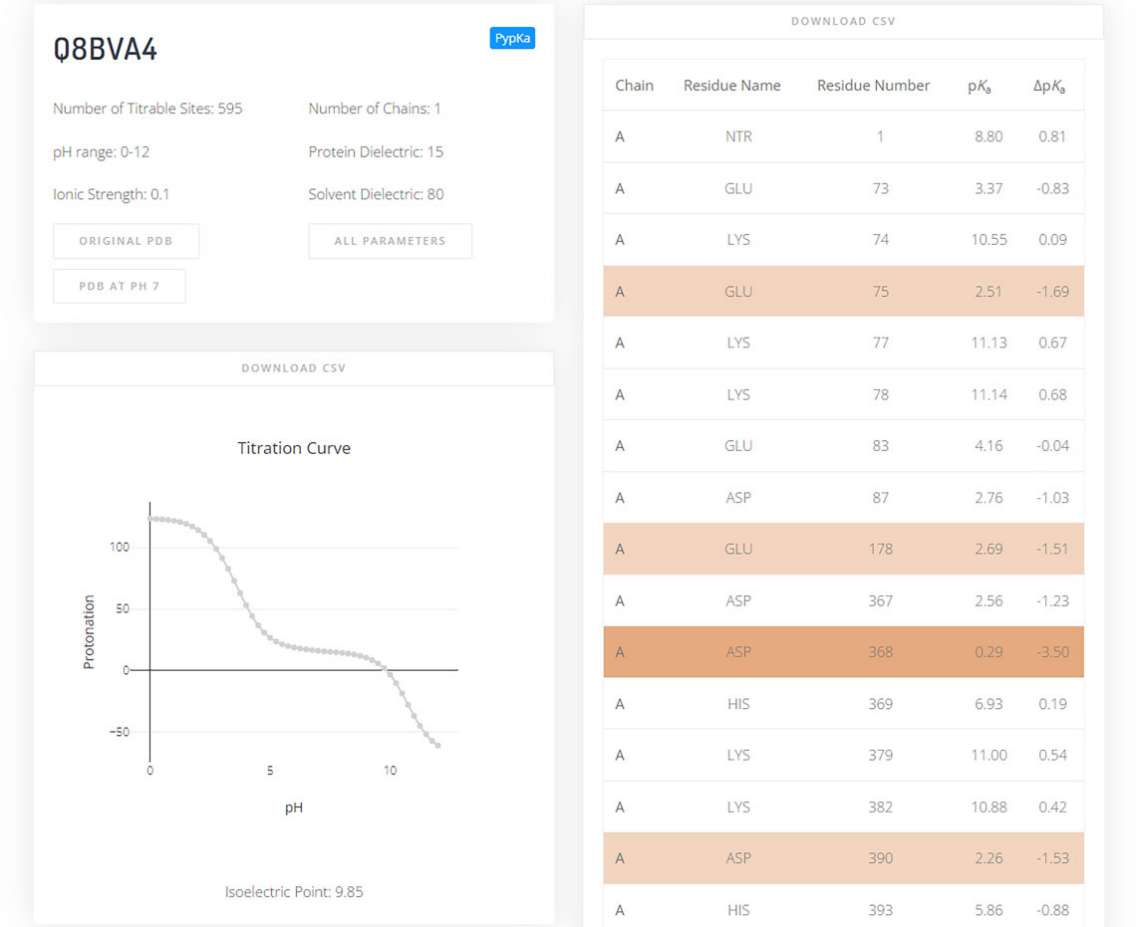
Example of a PypKa server output. On the top left a small informative card displays details about the system and calculation parameters. In this card, there is also a link to download the pdb file with a representative protonation state. A titration plot is shown below in which the isolectric point is highlighted. A table with the calculated p*K*_a_ values is shown on the right side. In this table, residues with a |Δp*K*_a_| values greater than 1.5 are highlighted in light orange and residues with a |Δp*K*_a_| greater than 2.5 are highlighted in darker orange. The user can download the titration curve and the p*K*_a_ values as csv files.

Once the results are returned to the front end, the user may visualize and download them. An example of the output page shown in the server is illustrated in Figure 2. The protein’s isoelectric point value is shown and can also be inferred by inspecting the total titration curve plot. This titration curve can be downloaded as a csv file, as well as the table with the p*K*_a_ estimations. In this table, a colour code has been used to help identify residues that are markedly shifted compared to their reference p*K*_a_ values in water. As for downloadable output structures, the user can obtain a formatted pdb file with the titratable protons placed in the most likely configuration according to the p*K*_a_ calculations which include proton tautomerism. The nomenclature for the output file includes the same force field input options (AMBER, CHARMM and GROMOS). For the calculations performed with pKAI, the titration curve, and most likely protonation states are approximated from the p*K*_a_ predictions, while with PypKa they are directly obtained from the Monte Carlo simulations.

Unfortunately, there are PypKa jobs that fail, usually due to missing atoms that PDB2PQR cannot reconstruct or repair. While pKAI is able to handle most of these structures, users are strongly advised to inspect these failed structures and fix them manually or use third-party software. Another known issue is related to the size of the input structure. The current settings of the server limit the usage of PypKa to structures with<1500 residues, and for larger systems, pKAI is automatically used.

## Conclusion

Nowadays, there are several computational methods to predict p*K*_a_ values in proteins and/or to prepare biomolecular structures for molecular modelling pipelines, with some being available as web servers. Our PypKa server stands out from these methods by providing the ability to quickly retrieve results for structures from commonly used repositories and to run extremely large systems with ML-accelerated models while supporting the most popular MD force fields. Another valuable feature of this service is the REST API that allows users to integrate it into their existing scripts, protocols, as well as other web services to quickly have access to p*K*_a_ values, isoelectric points, and titration curves.

## Data Availability

The web server is freely available at pypka.org and does not require user registration or login. The REST API can be accessed at api.pypka.org. The code for both the front end and the back end is hosted in GitHub and can be inspected at mms-fcul/PypKa-Server-Front and mms-fcul/PypKa-Server-Back, respectively. Permanent DOIs: https://doi.org/10.5281/zenodo.10878258 and https://doi.org/10.5281/zenodo.10878237.
